# A Random Thought on Nitrous Oxyd

**Published:** 1867-09

**Authors:** Geo. Watt


DENTAL REGISTER.

VOL. XXI.] SEPTEMBER, 1867. [No. 9.

### Original Communications.

### A RANDOM THOUGHT ON NITROUS OXYD.

BY GEO. WATT.

(For the Mad River Valley Dental Society.)

MR. PRESIDENT AND GENTLEMEN: It was my intention to write at length on the subject you assigned me for this meeting; but an overwhelming amount of literary labor, much of it unexpected, has prevented. That it may appear that I have forgotten neither yourselves nor your appointment, I hastily pen a thought or two.

Allow me to say that I am more and more impressed with the importance of the subject, and more in favor of a judicious use of the gas than ever before. And never before did I feel so strongly, the importance of using only *pure nitrous oxyd.*

In our schoolboy days, when bad results followed the administration of "laughing gas," the explanation was that some temperaments could not bear it. Now, let it be remembered that *pure* nitrous oxyd agrees with all temperaments, and probably with all conditions of the constitution. But this must not be misunderstood. A patient may be afraid of the gas, or may dread the operation, or from various causes, may be unduly excited, at the time. Such patients are not apt to breathe freely, and, therefore, the blood is not decarbonized, and the system suffers. Then the apparatus may be so arranged as to make breathing difficult. The delivery tube may be too long, or the valves of the inhaler too contracted. Of course, the blood will not be duly oxygenated, and the patient is the worse for the inhalation. But when *pure* nitrous oxyd is inhaled as tranquilly and as easily as atmospheric air usually is, *it can disagree seriously with no one.*

I will not, in this paper, discuss its *modus operandi*, in producing anæsthesia. But it appears to be on principles totally different from those of chloroform and ether.

An important practical point, in using absolutely pure gas, is to avoid carrying the patient beyond the stage of complete anæsthesia. I do not recollect of having seen any thing in the books, or journals, to lead us to suspect that this is even possible. The general impression appears to be that the longer a patient breathes the gas, unmixed with air, the more profound the unconsciousness; and I can account for this only on the theory of not experimenting with pure gas. Even when pure gas has been obtained, till very recently, the experimenters only inhaled *one* pure breath of it, before commingling with it the carbonic acid and other deleterious agents contained in their expirations. Each breath, diminishing the quantity of oxygen, and increasing the carbonic acid, tended to bring on a state of asphyxia, in connection with anæsthesia. Patients thus smothered, may remain unconscious in proportion to the time spent in the process of suffocating them; suffocation, rather than anæsthesia, being the proper term to designate the process.

But when pure gas is freely breathed, the patient soon becomes unconscious; but by the time the superoxydized blood has become commingled with the general circulation, the breathing becomes less frequent and less full, the patient feeling no inclination to breathe more than enough to decarbonize the blood. Such a patient soon returns to consciousness, and may breathe the gas for an indefinite period, without mental or muscular disturbance. I have said for an indefinite period, simply meaning that I know not how long it may be thus breathed. I have breathed it so myself for ten minutes, being fully self-possessed, reading, making mathematical calculations, etc., requiring not only consciousness, but concentration of thought; and at the close of the experiment the muscles were as obedient to the will as usual. I have had similar experience with others.

Then, to produce perfect anæsthesia, the gas should be breathed freely at the start, and all admission of atmospheric air should be prevented. Many patients fail to breathe freely and fully through dread of the operation—fearing that it will be painful, or if not, that they will never return to consciousness. Some patients can not control themselves so as to aid the respiratory function, by voluntary effort. Their breathing, at best, may be feeble. With such, it is often difficult to produce full anæsthesia. They should be induced to practice full inspirations for a little while before trying to inhale the gas; as by the effort the air cells are expanded, the respiratory muscles are brought freely into play, and the patient usually begins the gas with correspondingly full inspirations. A patient once breathed twenty-one gallons of the gas, and at another sitting thirty, without being at all unconscious. By adopting the measures just recommended, he was brought to a state of complete unconsciousness, by inhaling two and a half gallons.

But it is fortunate that those patients who breathe slowly and feebly are not usually excitable. Hence, they are generally calm enough to sit still for an operation, even though conscious that it is about to be performed. The fact of consciousness is not proof of pain; for it has been long recognized that a severe operation may be performed while the patient does not suffer, though conscious of all that is going on. Any one familiar with the use of chloroform must have seen cases that prove this position.

This return to consciousness while breathing the pure gas, indicates that nitrous oxyd is not likely to supercede ether and chloroform for tedious operations, though I have known it highly successful in those requiring from five to fifteen minutes; but in the latter part of the operation, in each case, the patient was quite conscious, though quite indifferent, as to all that was in progress. *A conscious state, free from pain and prostration during operations,* is the goal to strive for. The man that reaches it will be crowned by the goddess of surgery, if there is such a personage. It has been more nearly reached by nitrous oxyd than by any other anæsthetic. The time is coming when a surgeon may oversee, or even perform the amputation of one of his own extremities, as composedly as he now pares his nails, and as free from pain.

And, in a short digression, allow me to state that it appears strange that nearly all the search after anæsthetics has been made among liquids. The air we breathe is a mixture of gases. The first used practical anæsthetic is a compound gas. The mucous membrane of the respiratory passages is adapted to gases, rather than to vapors. But, notwithstanding, the gases were abandoned in favor of the various ethers, which are liquid at ordinary temperatures. I believe it would be easy to prepare a mixture of gases but little, if any, more prostrating or nauseating than nitrous oxyd, and yet as lasting in its anæsthetic effects as ether or chloroform.

Having insisted on the purity of nitrous oxyd as a condition of success, it is well to inquire what agents are most like to contaminate it, why these are objectionable, and how we may avoid their presence.

The contaminating agents most likely to annoy us in practice are atmospheric air, vapor of water, chlorine, and nitric oxyd, or binoxyd of nitrogen.

The first of these annoys by the patient inhaling it along with the gas, the result being a failure to secure complete anæsthesia, the patient reaching that state referred to above, in which he will not breathe even the pure gas with sufficient rapidity to render him unconscious. An improved mouthpiece will remedy this difficulty.

Nitrous oxyd, as ordinarily preserved, becomes diluted by mixture with the vapor of water. This does not interfere with its purity in a therapeutic sense, nor does it ordinarily result in any inconvenience. When I find a patient is hard to influence, if a second operation is necessary, I endeavor to have gas very recently prepared, to meet the case. Thus far the difficulty is *dilution*, rather than adulteration.

But when chlorine is present, the consequences are likely to be serious. This gas, even when much diluted, is highly poisonous, causing, when breathed, great irritation of the air passages, as well as constitutional disturbance. If the nitrate of ammonia is contaminated by *sal ammoniac*, called sometimes muriate of ammonia, and chloride of ammonium, chlorine will be liberated when the salt is decomposed by heat. When there is any doubt, the salt should be tested. In the absence of test tubes, small vials will answer. What are called "homœopathic vials" will be convenient. A portion of the nitrate is to be dissolved in pure water. Dissolve also a small quantity of nitrate of silver in pure water. Pour the solution of the nitrate of ammonia gradually into that of the nitrate of silver. If any soluble chloride is present, a grayish white precipitate will be formed, which will render the solution milky in appearance at first, the precipitate gradually settling to the bottom. Chlorine being twice as soluble in water as nitrous oxyd, a moderate proportion of it may be washed out; but the only judicious and safe way is not to make it.

Unfortunately it has been the general belief that when pure nitrate of ammonia is used, pure nitrous oxyd will be obtained. This is a very serious mistake. At least three violent poisons may be generated from the pure nitrate. But the one most likely to be formed, the hardest to separate from the nitrous oxyd, and the most deleterious in its effects when inhaled, is the binoxyd of nitrogen or *nitric* oxyd.

This gas is colorless, and therefore invisible, is much lighter than nitrous oxyd, and far less soluble in water. If any of it is formed it is likely to pass into the gasometer. Unfortunately, some of our authors tell us that it, and all other impurities, are removed by passing the gas through water. To wash this out of nitrous oxyd, would be equivalent to washing sand out of sugar. If equal quantities of the two gases were commingled, and the mixture passed through water, by the time the water had absorbed all the *nitrous* oxyd, eighty-nine per cent. of *nitric* oxyd would still remain.

To separate nitric, from nitrous oxyd, some writers tell us to put sulphate of iron in the washers. But this is not satisfactory. When it is not the intention to form any nitric oxyd, and when we have no means of knowing, at the time, how much is made, how are we to know how much sulphate to use? Again, we are told to leave some atmospheric air in the gasometer. If nitric oxyd is formed, it will be changed to nitrous acid by the oxygen of the air, and this will be dissolved in the water. But how much air shall we leave in? If too much, our gas is *diluted*, and not reliable. If too little, we still have the poison.

But if nitric oxyd is formed, why is its presence so objectionable? Think, for a moment, of its nature and properties. It is composed of one equivalent of nitrogen united with two of oxygen. When exposed to atmospheric air, it takes two more equivalents of oxygen, and is changed to nitrous acid. This, in contact with water, is changed to nitric acid, by taking an equivalent of oxygen from the water. And who would not shrink from the thought of cauterizing the entire pulmonary mucous membrane with the vapors of nitric acid? But the nitric acid formed as above described, is brought in contact with this membrane, in its nascent state, being then as much more caustic than ordinary nitric acid, as ozone is more corrosive than oxygen. That nitric oxyd goes through these changes rapidly, may be demonstrated by a simple experiment in the hands of any one. Nitric oxyd is colorless. When dilute nitric acid is poured on copper, or a similar metal, this gas rises in small bubbles. These have scarcely escaped into the air till orange colored, or red fumes are seen, showing that the change to nitrous acid has already taken place.

The only proper and safe way, then, is not to generate the nitric oxyd. And this involves the control of the temperature at which the nitrate is decomposed. I am constantly becoming more careful on this point. The heat regulator of Sprague's apparatus very nearly accomplishes the desired result in this direction. That is, when illuminating gas is used in heating. I am not familiar with the action of his regulator for Kerosene heaters. It is altogether impracticable to regulate the heat by the eye. No inexperienced person would believe how small the flame must be toward the close of the process. When Dr. Taft told us, in the REGISTER, that nitrous oxyd should be prepared and used only by a good chemist, or by one trained by a good chemist, I thought he had slightly overstated the matter; but, as he is usually so correct that it is dangerous to differ with him, I kept quiet. I am quite convinced that he was "right in his head." I would pay a good chemist a liberal fee for just a little more information on nitrous oxyd, rather than work it out myself, by a tedious process of experiments. Since our last meeting I have tried nearly a hundred experiments to find out a single fact stated in this paper. I suspected it to be a fact then; but I didn't *know* it to be one.

For the various reactions that may take place in decomposing nitrate of ammonia by heat, I refer you to a brief article in the December number of the REGISTER, which was republished in the *Cosmos*. Since writing that I have received many letters of inquiry, many of which I have not had time to answer. Some of them tell me they have no heat regulator, but they "have a boy to watch it," that their heater is small, and they don't have a large flame,—they had to get their apparatus because their competitors used gas—

and they had "a real good gasometer," and did'nt I think they were safe in using it, and so on *ad infinitum.* One day I got a number of these letters. I went to sleep at night while my imagination was carrying me back to the days of James Watt. I thought a young engineer wrote to him that he had put up a first rate steam engine, but could not afford a patent steam gauge. So to make it safe he put a small fireplace under the boiler, and got wet shavings for kindling, used only green wood, and hired a very lazy fireman, and told him he needn't fire up very vigorously, and now, didn't he think it was safe for the hands to work above it in the factory. I am sometimes overrun with such inquiries from those who try thus to coax from me a *quasi* endorsement of that which their own consciences condemn.

